# Fine Particulate Matter Leads to Unfolded Protein Response and Shortened Lifespan by Inducing Oxidative Stress in *C. elegans*

**DOI:** 10.1155/2019/2492368

**Published:** 2019-12-07

**Authors:** Yunli Zhao, Ling Jin, Yuxin Chi, Jing Yang, Quan Zhen, Huazhang Wu

**Affiliations:** ^1^Department of Preventive Medicine, Bengbu Medical College, Bengbu 233030, China; ^2^School of Life Science, Anhui Province Key Laboratory of Translational Cancer Research, Bengbu Medical College, Bengbu, China

## Abstract

Oxidative stress has been proven as one of the most critical regulatory mechanisms involved in fine Particulate Matter- (PM_2.5_-) mediated toxicity. For a better understanding of the underlying mechanisms that enable oxidative stress to participate in PM_2.5_-induced toxic effects, the current study explored the effects of oxidative stress induced by PM_2.5_ on UPR and lifespan in *C. elegans*. The results implicated that PM_2.5_ exposure induced oxidative stress response, enhanced metabolic enzyme activity, activated UPR, and shortened the lifespan of *C. elegans*. Antioxidant N-acetylcysteine (NAC) could suppress the UPR through reducing the oxidative stress; both the antioxidant NAC and UPR inhibitor 4-phenylbutyric acid (4-PBA) could rescue the lifespan attenuation caused by PM_2.5_, indicating that the antioxidant and moderate proteostasis contribute to the homeostasis and adaptation to oxidative stress induced by PM_2.5_.

## 1. Introduction

Oxidative stress has been described as the disturbances in the redox homeostasis resulting from increased reactive oxygen species (ROS) production or attenuated antioxidant defense systems [1, 2]. Oxidative stress usually leads to damage as free radicals can interact with cell components such as DNA, lipid, and protein [3, 4]. As a common denominator of toxicity, oxidative stress can be induced almost by all types of toxicants. The free radicals can be produced and aggregated in the endoplasmic reticulum (ER) and mitochondria, which may lead to the accumulation of unfolded/misfolded proteins and have extensive influence on homeostasis and function [5–9]. Organisms have developed excellent defense mechanisms to prevent damage caused by environmental stresses, such as signaling pathways that resolve aberrant proteins known as unfolded protein response (UPR) [10, 11]. Previous studies have shown that xenobiotics can activate UPR signaling network *in vivo* and *in vitro* [12–14].

The model system *C. elegans* was extensively used for toxicology researches because of its distinct advantages, such as short lifecycle and ease of culture [15, 16]. In *C. elegans*, *hsp-4* encodes endoplasmic reticulum chaperone BiP homolog and *hsp-6* encodes a mitochondrial-specific chaperone, which was believed to be involved in the endoplasmic reticulum (UPR^ER^) and mitochondrial unfolded protein response (UPR^mt^), respectively [17, 18].

As a major compound of the ambient air, particulate matter (PM) has become a widespread environmental concern for its health threat [19, 20]. Fine particulate matter (PM_2.5_, particles with a diameter of 2.5 *μ*m or less) can not only reach parts of the respiratory tract but also penetrate deeply into the lung alveoli and enter the bloodstream due to their small size [21, 22]. Numerous studies have demonstrated that long- and short-term exposures to PM_2.5_ are related to respiratory disease and cardiovascular morbidity [22–24].

Due to the adverse health effects of PM_2.5_, scientists have done extensive researches on the molecular mechanisms underlying the toxicity of PM_2.5_, and oxidative stress was considered as one of the primary mechanisms implicated in PM_2.5_-mediated toxicity [25, 26]. Previous studies have proven that PM_2.5_ exposure could generate excessive ROS and thus decrease antioxidant enzyme activities, resulting in oxidative stress in cells [27, 28]. Besides, chronic inhalation exposure to PM_2.5_ triggered two distinct UPR signaling pathways in mice [21, 29]. We speculate that UPR may coordinate proteostasis responses in regulating oxidative stress tolerance following PM_2.5_ exposure. But the association between UPR and oxidative stress still remains unclear, and the causal explanation for this interaction may require further investigation.

In the present study, we found that PM_2.5_ exposure induced oxidative stress processes, enhanced metabolic enzyme activity, activated UPR, and shortened the lifespan of *C. elegans*. Our results not only reveal the role of UPR in response to oxidative stress following PM_2.5_ exposure and the relationship between oxidative stress and UPR activation but also provide important information for protection against the toxic effect of PM_2.5_.

## 2. Materials and Methods

### 2.1. PM_2.5_ Sampling and Concentration Analysis

PM_2.5_ was collected by medium-volume TSP samplers with PM_10_ and PM_2.5_ separators (TH-150 C; Wuhan Tianhong Environmental Protection Industry Co., Ltd., China). Samples of PM_2.5_ were collected in the campus of Bengbu Medical College in Anhui Province of China, a place far away from the highway or manufacturing district. Continuous sampling was performed for 24 hours at a flow rate of 100 L/min on quartz microfiber filters for a toxicity assay and PTFE (polytetrafluoroethylene) microfiber filters for the determination of elemental composition (90 mm; Wuhan Tianhong Environmental Protection Industry Co., Ltd., China). The sampling was carried out from December 2018 to January 2019. To eliminate any adsorbed organic compounds, the filters were pretreated at 600°C for 2 h before collection. The PM_2.5_ samples were extracted by cutting filters into small pieces and immersing into deionized water, followed by sonicating for 40 min. Then, the sample was freeze dried and stored at -20°C. The particles were weighed and resuspended with K buffer or deionized water before use.

The concentrations of 16 elements were detected by inductively coupled plasma atomic emission spectroscopy (ICP-MS, GE Co., Ltd., USA). The PAHs (polycyclic aromatic hydrocarbons) were extracted with dichloromethane and were analyzed using gas chromatography coupled to a mass spectrometer (GC-MS, Agilent, CA, USA). The concentrations of 16 elements and PAHs analyzed in the PM_2.5_ samples are listed in Tables [Supplementary-material supplementary-material-1] and [Supplementary-material supplementary-material-1] in the Supplementary Materials.

### 2.2. Strains and Maintenance

Strains used in this study were gifts from the Caenorhabditis Genetics Center (CGC) and maintained on standard nematode growth medium (NGM) seeded with *Escherichia coli* OP_50_ as described in reference [30]. The transgenic strains CF1553 (*muIs84 [(pAD76) sod-3p::GFP+rol-6(su1006)]*) and CL2166 (*dvIs19 [pAF15(gst-4::GFP::NLS)]*) were used to visualize the expressions of the oxidative stress-resistance-related proteins SOD-3 and GST-4 illustrating the inducible oxidative stress in PM_2.5_-exposed worms [31]. Transgenic strains SJ4005 (*zcIs4 [hsp-4::GFP] V*) and SJ4100 (*zcIs13 [hsp-6::GFP]*) were used as the indicators for the unfolded protein response in the endoplasmic reticulum UPR (UPR^ER^) and mitochondrial UPR (UPR^mt^) [32], respectively.

### 2.3. Exposure Methods

For exposure, PM_2.5_ was diluted into different concentrations with K buffer (53 mmol/L NaCl and 32 mmol/L KCl) and added into a 96-well plate; then, L4 larvae worms were transferred with a platinum picker. Worms were treated from L4 larvae for 24 hours at 20°C in a 96-well plate with OP_50_ as food, with 30 worms per well and 4 parallel wells for one concentration. Synchronized L4 larvae were obtained by culturing the synchronized eggs at 20°C for 36 hours fed with OP_50_. To obtain synchronized eggs, gravid nematodes were washed by K buffer followed by a bleaching mixture (0.45 mol/L NaOH, 2% HOCl) [33].

### 2.4. ROS Induction Assessment

For ROS induction assessment, worms were washed into the centrifugal tube and CM-H_2_DCFDA (C6827, Invitrogen/Molecular Probes) was added to a final concentration of 1 *μ*M followed by incubating at 20°C for 5 hours in the dark. After treatment with CM-H_2_DCFDA, worms were mounted onto agar pads for ROS production detection [34]. The fluorescence signal was observed under a fluorescence microscope (Zeiss, Axio Observer Z1, Germany) **(**excitation wavelength: 480 nm; emission wavelength: 510 nm), and the intensities of the relative fluorescent units (RFU) in the intestine were measured and quantified by the ImageJ program (NIH, Bethesda, MD). Three individual repetitions for each condition were performed, and at least 20 nematodes were measured per replication.

### 2.5. Measurement of Oxidative Stress Markers

After exposure, worms were collected and washed with K buffer, then precooled lysis buffer (pH 7.4, 0.01 mol/L Tris-HCl, 0.0001 mol/L EDTA-2Na, 0.01 mol/L saccharose, and 0.8% NaCl) was added and the mixture was transferred to a glass homogenizer for homogenizing. After homogenizing, the mixture was transferred to 1.5 mL Eppendorf tubes and centrifuged at 3000 × g for 10 min, and the supernatant was collected for the use of LDH and MDA measurement. The Bradford method was used for protein concentration with bovine serum albumin (BSA) as the standard.

The intracellular lactate dehydrogenase (LDH) release was used to reflect cell membrane integrity [35, 36]. LDH release was measured with a commercial assay kit (Nanjing Jiancheng Bioengineering Institute, China) and monitored using the Multiskan Ascent (BioTek Instruments, Inc., USA) with the absorbance at the wavelength of 490 nm and normalized by protein concentrations. The LDH leakage (% of control) was presented as the percentage of control, with K buffer as blank control.

Malondialdehyde (MDA) as a unique end-product of lipid peroxidation was usually used to represent the extent of lipid peroxidation reactions because lipid peroxidation often occurs while animals were injured [37]. MDA was measured using the MDA assay kit (Nanjing Jiancheng Bioengineering Institute, China) following the manufacturer's instructions. The absorbance of the supernatant was detected by a microplate reader (BioTek Instruments, Inc., USA) at 532 nm and normalized by protein concentrations.

### 2.6. The Activities of Antioxidant Enzymes

After exposure, worms were collected and washed with K buffer. After that, precooled lysis buffer was added and transferred to a glass homogenizer for homogenizing. Then, the homogenate was transferred to 1.5 mL Eppendorf tubes and centrifugated at 3000 × g for 10 min, and the supernatant was used to evaluate the activities of the antioxidant enzymes catalase (CAT) and glutathione peroxidase (GSH-Px) within 24 hours. The Bradford method was used for protein concentration with bovine serum albumin (BSA) as the standard.

The activities of CAT and GSH-Px were evaluated with a commercial assay kit (Nanjing Jiancheng Bioengineering Institute, China) and the absorbance was monitored using a spectrophotometer (BioTek Instruments, Inc., USA) at the wavelengths of 450 nm and 405 nm, respectively. The activities of CAT and GSH-Px were normalized by protein concentrations and presented as the percentage of control.

### 2.7. Lifespan Measurement

After being exposed to PM_2.5_ in the 96-well plate for 24 hours, worms were transferred to 3.5 cm NGM plates with the *E. coli* OP_50_ for lifespan measurement. Worms were transferred to fresh plates daily for the first 5 days and every 2 days after that. Survival was monitored every day and worms with no response to touches with a platinum wire and no pharyngeal pumping were defined dead [16]. Survival curves were plotted by the Kaplan-Meier method using SPSS 23.0 (IBM, USA), and mean lifespans were calculated for statistical analyses. Experiments were performed in triplicate and more than 100 worms were scored for each experiment analysis.

### 2.8. Quantitative RT-PCR

RNA was isolated using Trizol (Invitrogen, Carlsbad, CA) and extracted with chloroform, and cDNA was synthesized by reverse transcription with random primers on total RNA (Takara, Japan). The expression of *hsp-4* and *hsp-6* was measured using SYBR® Green by Quantitative RT-PCR (qRT-PCR) and normalized with *tba-1* and *act-1* [38]. Primers used for qRT-PCR were listed in [Supplementary-material supplementary-material-1] in the Supplementary Materials.

### 2.9. Statistical Analysis

Statistical evaluation was conducted with the SPSS 23.0 (IBM, USA). One-way ANOVA was used to compare the difference among different exposure conditions and *P* values less than 0.05 or 0.01 were considered to be statistically significant.

## 3. Results and Discussion

### 3.1. PM_2.5_ Induced Oxidative Stress in *C. elegans*

**Figure 1:**
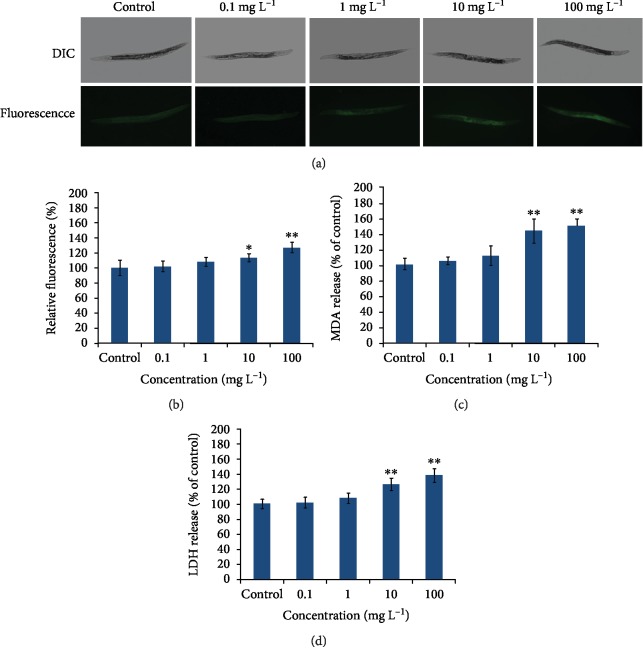
Signs of oxidative stress in *C. elegans* induced by PM_2.5_. (a) Image shows the fluorescence in the intestine of *C. elegans*. (b) Comparison of the reactive oxygen species (ROS) induced by PM_2.5_. (c) Effects of PM_2.5_ exposure on the production of malondialdehyde (MDA). (d) Effects of PM_2.5_ exposure on lactate dehydrogenase (LDH) release. The error bars represent the standard deviation of measurements. ^∗^*P* < 0.05 vs. control; ^∗∗^*P* < 0.01 vs. control.

To explore the potential role of PM_2.5_ in oxidative stress, three oxidative stress markers (reactive oxygen species (ROS), lactate dehydrogenase (LDH), and malondialdehyde (MDA)) were used to assess oxidative stress in response to PM_2.5_ in *C. elegans*. As shown in Figure 1, signs of oxidative stress in *C. elegans* were observed when exposed to PM_2.5_ at the concentrations of 10 mg/L and 100 mg/L (Figures 1(a) and 1(b)). PM_2.5_ exposure induced more intestine ROS production, elevated LDH release, and MDA generation, and these inductions were proportional to the concentration of PM_2.5_ when the exposure concentrations were higher than 10 mg/L (Figure 1). These results suggested that PM_2.5_ was able to increase oxidative stress in *C. elegans*.

### 3.2. PM_2.5_ Altered the Activities of Antioxidant Enzymes in *C. elegans*

**Figure 2:**
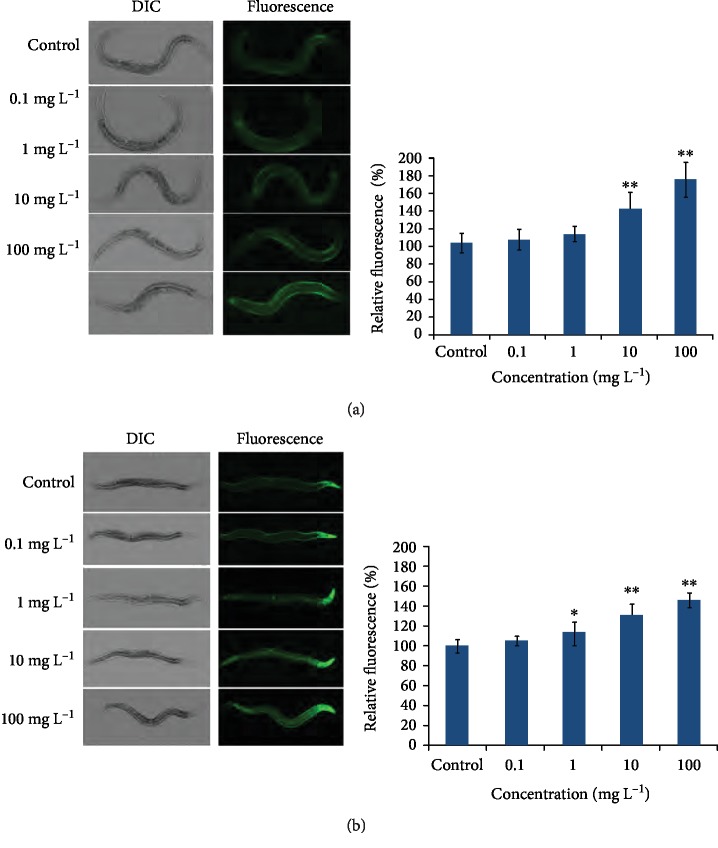
PM_2.5_ treatment enhanced the activity of the antioxidant enzyme in *C. elegans*. (a) Image of the expression of *gst-4P::GFP* in CL2166 worms and quantification of GFP fluorescence. (b) Image of the expression of *sod-3P::GFP* in CF1553 worms and quantification of GFP fluorescence. The error bars represent the standard deviation of measurements. ^∗^*P* < 0.05 vs. control; ^∗∗^*P* < 0.01 vs. control.

Under oxidative conditions, animals would activate some oxidative stress response enzymes to defend against oxidative stress and maintain the balance of the oxidative-antioxidative system. The transgenic nematodes CL2166 and CF1553 were treated as a transgenic reporter to monitor the inducible glutathione S-transferase (GST-4) and superoxide dismutase (SOD-3) expression. As shown in Figure 2, the expressions of GST-4 and SOD-3 were both significantly elevated with the increasing concentration of PM_2.5_ (Figure 2).

**Figure 3:**
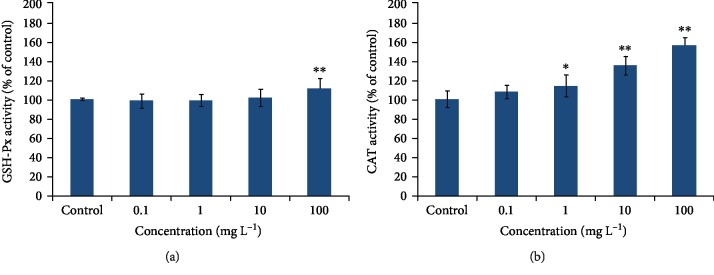
PM_2.5_ treatment increased the activities of GSH-Px (a) and CAT (b) levels in *C. elegans*. The error bars represent the standard deviation of measurements. ^∗^*P* < 0.05 vs. control; ^∗∗^*P* < 0.01 vs. control.

The activities of antioxidant enzymes were also observed by determining the activities of glutathione peroxidase (GSH-Px) and catalase (CAT) levels. Results demonstrated that *C. elegans* enhanced the antioxidant defenses by increasing the activities of antioxidant enzymes, as the activities of GSH-Px and CAT were both evaluated in PM_2.5_-exposed *C. elegans* (Figure 3). As shown in Figure 3(a), the activities of GSH-Px were significantly enhanced in worms exposed with PM_2.5_ at 100 mg/L compared to the untreated ones (*P* < 0.01). In addition, the activities of CAT also increased after having been exposed to PM_2.5_ at 1 mg/L (*P* < 0.05), 10 mg/L, and 100 mg/L (*P* < 0.01). Taken all together, these data suggested that the expressions of antioxidant enzymes were induced or activities were enhanced to eliminate the excessive oxidation generated in PM_2.5_-exposed nematodes.

### 3.3. PM_2.5_ Activated Unfolded Protein Response in *C. elegans*

**Figure 4:**
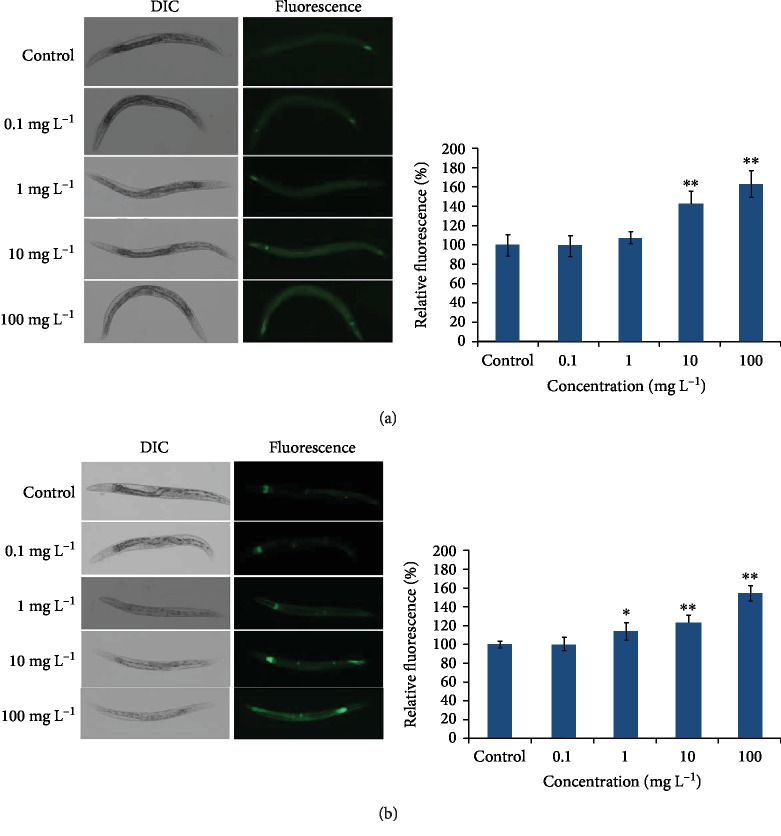
PM_2.5_ activated unfolded protein response in *C. elegans*. (a) Image of the expression of *hsp-6::GFP* in SJ4100 (*zcIs13 [hsp-6::GFP]*) worms and quantification of GFP fluorescence. (b) Image of the expression of *hsp-4::GFP* in SJ4005 (*zcIs4 [hsp-4::GFP] V*) worms and quantification of GFP fluorescence. The error bars represent the standard deviation of measurements. ^∗^*P* < 0.05 vs. control; ^∗∗^*P* < 0.01 vs. control.

The unfolded protein response (UPR) induced by PM_2.5_ was detected using the following transgenic strains: SJ4005 (*zcIs4 [hsp-4::GFP] V*) for UPR^ER^ and SJ4100 (*zcIs13 [hsp-6::GFP]*) for UPR^mt^. As shown in Figure 4, after having been exposed to PM_2.5_ at different concentrations, the GFP fluorescence intensities in *hsp-4P::GFP* and *hsp-6P::GFP* were both enhanced (Figure 4), indicating that both ER and mitochondrial UPR were activated in PM_2.5_-exposed animals. The *hsp-4* and *hsp-6* mRNA expressions were significantly elevated in PM_2.5_-exposed animals compared with control ([Supplementary-material supplementary-material-1]). These findings suggested that PM_2.5_ could induce both UPR^ER^ and UPR^mt^ in *C. elegans*.

### 3.4. ROS Scavenger NAC Could Suppress the UPR^mt^ and UPR^ER^ Activated by PM_2.5_ through Reducing the Oxidative Stress in *C. elegans*

**Figure 5:**
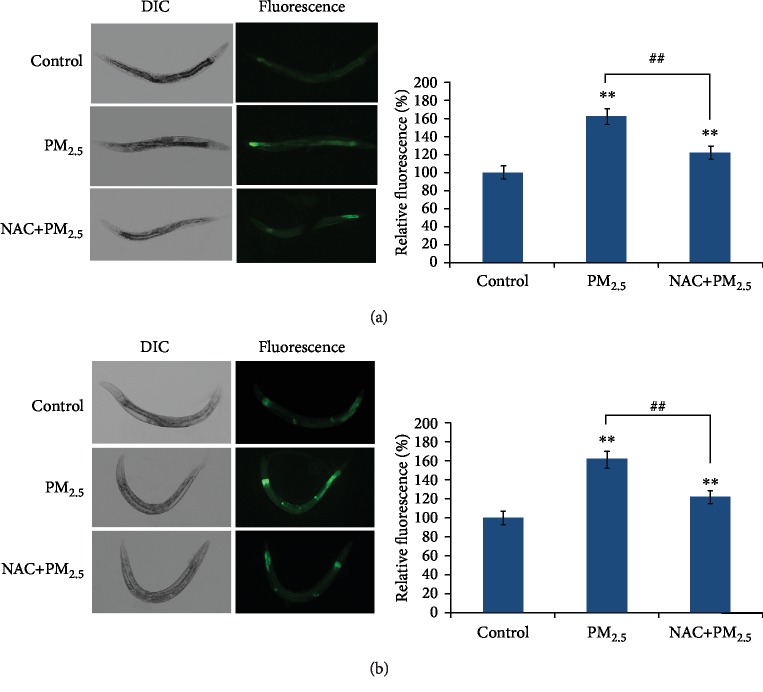
Antioxidant NAC could suppress the UPR^mt^ and UPR^ER^ activated by PM_2.5_. (a) Image of the expression of *hsp-6::GFP* in SJ4100 (*zcIs13 [hsp-6::GFP]*) worms and quantification of GFP fluorescence. (b) Image of the expression of *hsp-4::GFP* in SJ4005 (*zcIs4 [hsp-4::GFP] V*) worms and quantification of GFP fluorescence. The error bars represent the standard deviation of measurements. The exposure concentration of PM_2.5_ was 100 mg/L, and 2.5 mM NAC was added simultaneously. ^∗^*P* < 0.05 vs. control; ^∗∗^*P* < 0.01 vs. control; ^##^*P* < 0.01 vs. PM_2.5_.

To investigate whether the UPR^mt^ and UPR^ER^ activated by PM_2.5_ was the consequence of oxidative stress, we then used ROS scavenger NAC to ameliorate the oxidative state in PM_2.5_-exposed worms. According to the results above, 100 mg/L was chosen for exposure concentration of PM_2.5_ and 2.5 mM NAC was added at the same time. Results indicated that less GFP fluorescence was induced in worms exposed to PM_2.5_ and NAC simultaneously than those merely exposed to PM_2.5_, but this was still slightly higher than in those under normal conditions (Figure 5), indicating that the UPR^mt^ and UPR^ER^ induced by PM_2.5_ was not merely through oxidative damage. Analysis of mRNA expression for *hsp-4* and *hsp-6* received the same results ([Supplementary-material supplementary-material-1]). These findings supported the hypothesis that the UPR induced by PM_2.5_ may be the direct consequence of oxidative stress.

### 3.5. Fine Particle Matter Reduced the Lifespan of *C. elegans*

**Figure 6:**
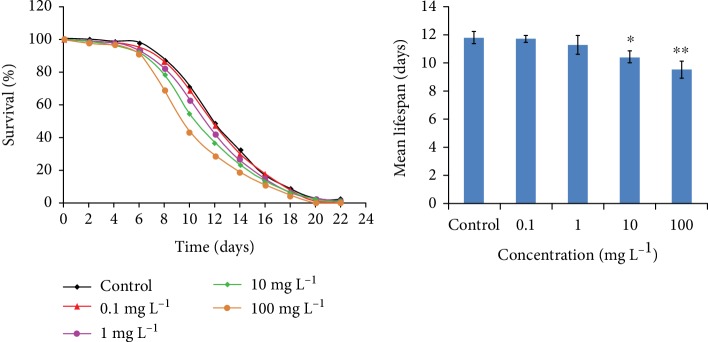
Effects of PM_2.5_ on the lifespan of *C. elegans*. The error bars represent the standard deviation of measurements. ^∗^*P* < 0.05 vs. control; ^∗∗^*P* < 0.01 vs. control.

As organisms are usually short-lived under an oxidative state [39], the lifespans of worms under normal and PM_2.5_-exposed conditions were tested. No significant influences on the median lifespan were observed in worms exposed to the low dose of 0.1 mg/L, but nematodes exposed to 10 mg/L and 100 mg/L PM_2.5_ had significantly shorter lifespans compared with untreated worms (Figure 6), which was consistent with previous results of oxidative stress. These results indicated that PM_2.5_ exposure reduced the lifespan of *C. elegans*.

### 3.6. Antioxidant NAC and UPR Inhibitor 4-PBA Could Attenuate the Lifespan Reduction Phenotype Caused by PM_2.5_ in *C. elegans*

**Figure 7:**
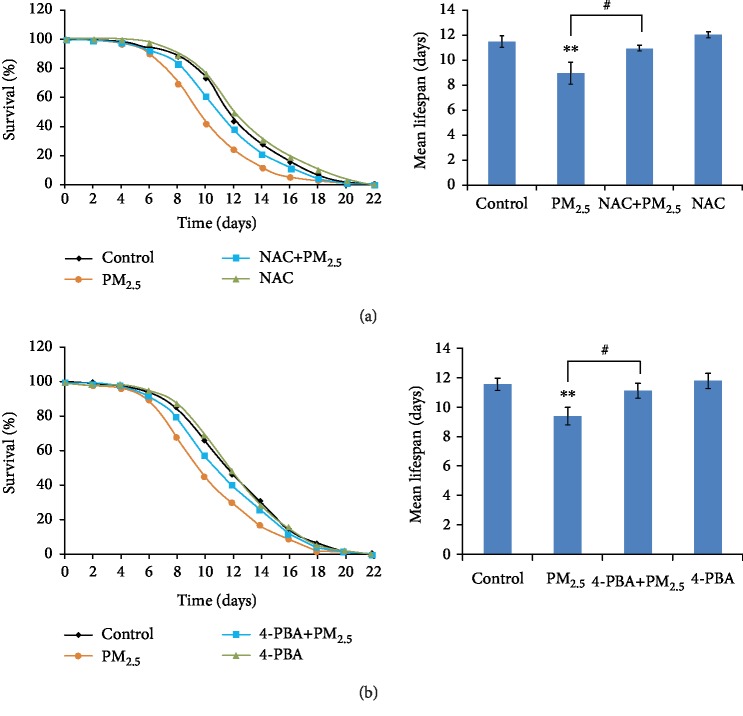
Antioxidant NAC and UPR inhibitor 4-PBA could attenuate the lifespan reduction phenotype caused by PM_2.5_ in *C. elegans*. The error bars represent the standard deviation of measurements. The exposure concentration of PM_2.5_ was 100 mg/L, and NAC and 4-PBA were used at 2.5 mM and 5 mM, respectively. ^∗^*P* < 0.05 vs. control; ^∗∗^*P* < 0.01 vs. control; ^#^*P* < 0.05 vs. PM_2.5_.

Previous studies demonstrated that resistance to oxidative damage increased the life of *C. elegans* [39], so we reasoned that if nematodes exposed to PM_2.5_ lived shorter lifespans because of the oxidative stress, then supplementation with antioxidant NAC could alleviate the short-lifespan phenotype in PM_2.5_-exposed worms. As expected, when worms were exposed to the antioxidant NAC (2.5 mM) and PM_2.5_ (100 mg/L) simultaneously, the mean lifespans were significantly extended compared to those exposed only to PM_2.5_ (Figure 7(a)), suggesting that the short-lifespan phenotype was a consequence of increased oxidative stress induced by PM_2.5_. Similar phenomena were found when 5 mM 4-phenylbutyric acid was used to attenuate the UPR response in PM_2.5_-exposed nematodes (Figure 7(b)), which means that the short-lifespan phenotype was correlated with UPR response induced by PM_2.5_. As the NAC has a slight effect of extending the lifespan of worms and 4-PBA has no effects on the lifespan (Figure 7), these results thus indicate that lifespan reduction in PM_2.5_-exposed nematodes was mediated by oxidative stress and UPR.

## 4. Conclusion

Oxidative stress acts as a response to the unfriendly environment and numerous toxicants in most organisms. In this study, our results were consistent with this viewpoint as ROS was produced in a dose-dependent manner in PM_2.5_-exposed worms, and LDH and MDA also increased by the same way (Figure 1). Organisms also have a defense mechanism against the adverse environment, and the detoxification response to xenobiotics was the vital one. SOD, CAT, GSH-Px, and glutathione S-transferase are important antioxidants that diminish oxidative stress through destroying the superoxide radical (O_2_^−^) or detoxifying H_2_O_2_ [40]. These antioxidative enzymes usually were induced in response to oxidative stress when organisms were under unfriendly conditions. In *C. elegans*, SOD-3, CAT, GSH-Px, and GST-4 act as antioxidative enzymes in diminishing the overproduced oxidative stress. The analytic results of the transgenic strains containing SOD-3::GFP and GST-4::GFP showed that SOD-3 and GST-3 were induced after exposure to PM_2.5_ (Figure 2) and the activities of CAT and GSH-Px were also increased after PM_2.5_ exposure, suggesting more antioxidants were induced to eliminate oxidative damage in *C. elegans* under PM_2.5_-exposed conditions. But this result does not agree with the study *in vitro*, as the LDH release is an indicator of the loss of cell membrane integrity, and an increase of LDH release usually leads to cell lysis and cell death [27, 36]; however, we still found enhanced CAT and GSH-Px activities in worms as LDH increased (Figure 3). We speculate that this may be due to organisms having a complex antioxidant defensive system in responding to oxidative stress. When the body deals with oxidative damage, the organism attempted to clean excess free radicals through increasing the activities of antioxidants in order to maintain a new balance of oxidation and antioxidation, hence the LDH and antioxidants were all increasing in PM_2.5_-treated worms. The responses to oxidative stress were inconsistent between cells and worms—maybe worms respond to stress as a whole with complex mechanisms, but the cells just act as an individual.

Oxidants can be generated and restored in the ER and mitochondria, which may lead to the accumulation of unfolded/misfolded proteins and vice versa [5]. Signaling pathways that resolve unfolded/misfolded proteins are called unfolded protein response (UPR) [11]. Based on this theory, we assume that PM_2.5_ may induce UPR in the ER and mitochondria. Transgenic strains SJ4005 (*zcIs4 [hsp-4::GFP] V*) and SJ4100 (*zcIs13 [hsp-6::GFP]*) were used as indicators for the unfolded protein response in the ER (UPR^ER^) and mitochondria (UPR^mt^). Our results showed that PM_2.5_ could induce the expression of *hsp-4::GFP* and *hsp-6::GFP* indicating that UPR^ER^ and UPR^mt^ were activated by PM_2.5_. In order to clarify whether UPR^ER^ and UPR^mt^ alleviate with the decrease of oxidative stress level, antioxidant NAC [41] was used to reduce oxidants induced by PM_2.5_. When worms were exposed to PM_2.5_ and NAC simultaneously, the fluorescence intensity in SJ4005 (*zcIs4 [hsp-4::GFP] V*) and SJ4100 (*zcIs13 [hsp-6::GFP]*) were both decreased compared to those in PM_2.5_-exposed ones (Figure 4), which suggest that the UPR^ER^ and UPR^mt^ activated by PM_2.5_ were associated with oxidative stress.

In biota, the rate of aging correlates with environmental factors and the lifespan usually declines under various stressors [42, 43]. *C. elegans* was a fit animal model for many stress analyses as its lifespan was short [43, 44]. In previous studies, environmental stresses including temperature, quality of nutrients, and toxicants could influence the lifespan of *C. elegans* [45, 46]. The same results were found with traffic-related fine particulate matter: both acute and prolonged exposure to PM_2.5_ could decline the lifespan and mean lifespan of *C. elegans* [16]. As for airborne PM_2.5_, we have found a similar tendency in the decline of lifespan, since worms lived a much shorter lifespan under PM_2.5_-exposed conditions (Figure 5). This is in accordance with the results showing that elevated total mortality and morbidity were associated with long-term exposure against PM [47]. As oxidative stress and ER stress are found associated with lifespan in *C. elegans* [39, 48], we next want to find out the interaction between the lifespan and oxidative stress or UPR, using antioxidant NAC and UPR inhibitor 4-PBA to remove oxidants or reduce ER stress at the same time worms exposed to PM_2.5_. The lifespan analysis demonstrated that NAC has a slight effect of extending the lifespan of worms, and 4-PBA has no significant effects on the lifespan (Figure 7); however, both NAC and 4-PBA could rescue the shortened lifespan when cotreated with PM_2.5_, indicating that the removal of oxidants and ER stress reduction could extend the lifespan of *C. elegans* and the shortening of life caused by PM_2.5_ was closely associated with oxidative stress and ER stress.

Taken together, our experiments demonstrated that fine Particle Matter (PM_2.5_) in the air pollution declines the lifespan of *C. elegans* through triggering UPR and elevated oxidative stress. NAC and UPR inhibitor 4-PBA could recover the lifespan shortened by PM_2.5_ through alleviating UPR and reducing the oxidative stress.

## Data Availability

All data used to support the findings of this study are included within the article.

## Supplementary Materials

Supplementary Materials Supplementary Table s1: metals detected in PM_2.5_ samples. The data are displayed as the mean ± SEM. Supplementary Table s2: organic compositions detected in PM_2.5_ samples. The data are displayed as the mean ± SEM. Supplementary Table s3: the primers used in this study. These primers were used for the verification of the expression of *hsp-4* and *hsp-6*. Figure s1: analysis of mRNA expression for *hsp-4* and *hsp-6*.
