# Supplementary material for: Fine Particulate Matter Leads to Unfolded Protein Response and Shortened Lifespan by Inducing Oxidative Stress in C. elegans
Source: Oxid Med Cell Longev. 2019 Dec 7;2019:2492368. doi: 10.1155/2019/2492368 (PMC6925806; doi:10.1155/2019/2492368)
Supplement: Supplementary Materials — Supplementary Table s1: metals detected in PM2.5 samples. The data are displayed as the mean ± SEM. Supplementary Table s2: organic compositions detected in PM2.5 samples. The data are displayed as the mean ± SEM. Supplementary Table s3: the primers used in this study. These primers were used for the verification of the expression of hsp-4 and hsp-6. Figure s1: analysis of mRNA expression for hsp-4 and hsp-6. [file 2492368.f1.doc]

**Supporting Information**

Table S1 Metals detected in PM_2.5_ samples

| Metals | Concentration (μg/mg) |
| --- | --- |
| Al | 0.122±0.053 |
| Si | 0.175±0.044 |
| S | 8.930±0.352 |
| Cl | 0.017±0.0057 |
| K | 0.226±0.029 |
| Ca | 0.523±0.025 |
| Ti | 0.035±0.041 |
| Cr | 0.005±0.002 |
| Mn | 0.018±0.004 |
| Fe | 2.117±0.121 |
| Ni | 0.003±0.003 |
| Cu | 0.020±0.007 |
| Zn | 0.093±0.010 |
| Br | 0.010±0.004 |
| Pb | 0.021±0.003 |

Table S2 Organic compositions detected in PM_2.5_ samples

| PAHs | Concentration (ng/mg) |
| --- | --- |
| acenaphthene | 0.367±0.077 |
| anthracene | 3.059±0.143 |
| benzo[ghi]perylene | 80.753±1.94 |
| benzo[a]pyrene | 31.259±2.995 |
| benzo[k]fluoranthene | 24.675±1.358 |
| benzo[b]fluoranthene | 35.570±3.095 |
| benzo[a]anthracene | 42.527±4.093 |
| chrysene, coronene | 0.238±0.036 |
| dibenz[a,h]anthracene | 13.970±1.304 |
| fluorene | 0.803±0.056 |
| fluoranthene | 54.217±3.384 |
| phenanthrene | 56.253±3.175 |

Table S3 primers used for qRT-PCR

| Gene | Forward primer (5’-3’) | Reverse primer (5’-3’) |
| --- | --- | --- |
| *hsp-4* | ATCGCCAACGATCAAGGA | CTCCGGATTGATTGTGAGC |
| *hsp-6* | TCGTGAACGTTTCAGCCAGA | CTCAGCGGCATTCTTTTCGG |
| *act-1* | GCTGGACGTGATCTTACTGATTACC | GTAGCAGAGCTTCTCCTTGATGTC |
| *tba-1* | TCAACACTGCCATCGCCGCC | TCCAAGCGAGACCAGGCTTCAG |


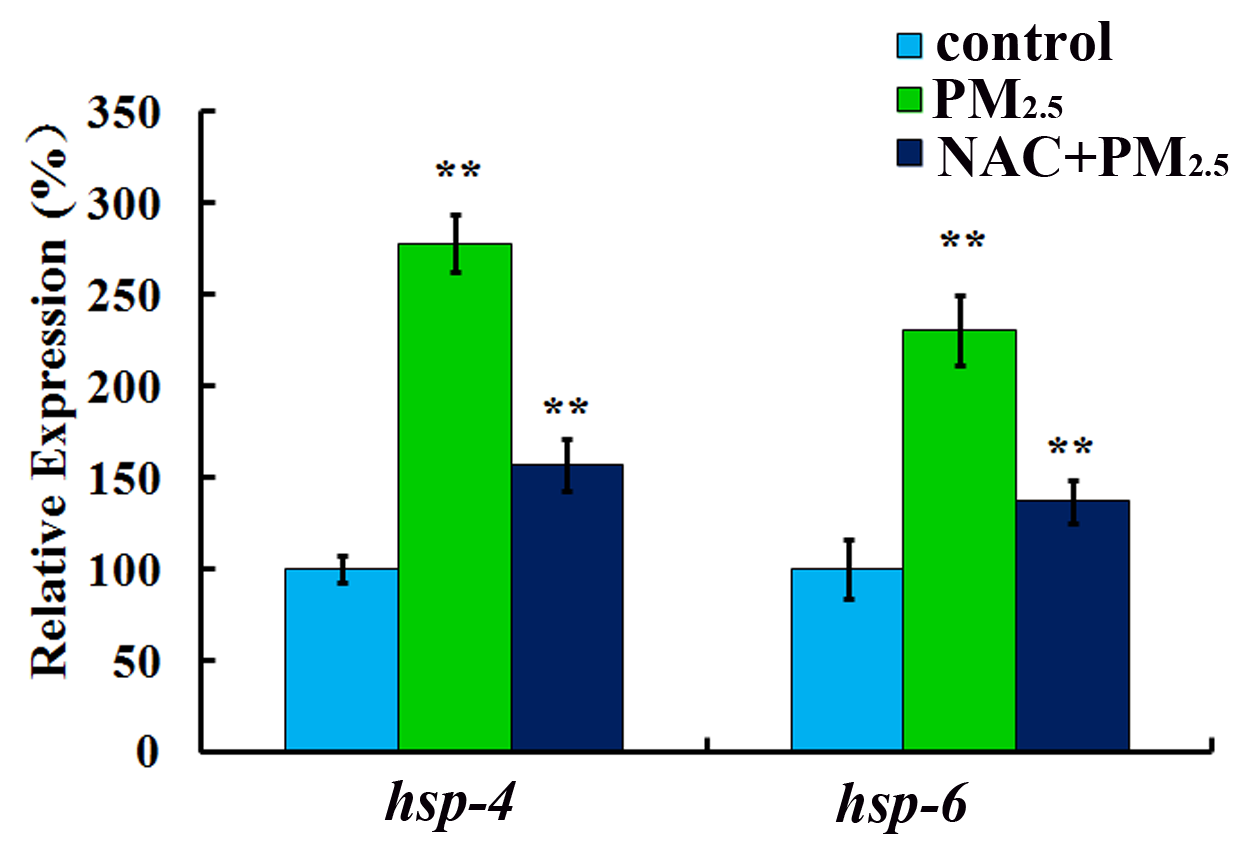


Figure s1: Analysis of mRNA expression for *hsp-4* and *hsp-6*
